# Pharmacologic Considerations in the Disposition of Antibodies and Antibody-Drug Conjugates in Preclinical Models and in Patients

**DOI:** 10.3390/antib8010003

**Published:** 2019-01-01

**Authors:** Andrew T. Lucas, Ryan Robinson, Allison N. Schorzman, Joseph A. Piscitelli, Juan F. Razo, William C. Zamboni

**Affiliations:** 1 University of North Carolina (UNC), Eshelman School of Pharmacy, Chapel Hill, NC 27599, USA; joseph_piscitelli@unc.edu (J.A.P.); juanrazo@email.unc.edu (J.F.R.); zamboni@email.unc.edu (W.C.Z.); 2 Division of Pharmacotherapy and Experimental Therapeutics, UNC Eshelman School of Pharmacy, University of North Carolina at Chapel Hill, Chapel Hill, NC 27599, USA; aschorz@email.unc.edu; 3 Lineberger Comprehensive Cancer Center, University of North Carolina at Chapel Hill, Chapel Hill, NC 27599, USA; ryan_robinson@med.unc.edu

**Keywords:** Antibody-drug conjugates, Pharmacology, Mononuclear phagocyte system, Pharmacokinetics, Therapeutic proteins

## Abstract

The rapid advancement in the development of therapeutic proteins, including monoclonal antibodies (mAbs) and antibody-drug conjugates (ADCs), has created a novel mechanism to selectively deliver highly potent cytotoxic agents in the treatment of cancer. These agents provide numerous benefits compared to traditional small molecule drugs, though their clinical use still requires optimization. The pharmacology of mAbs/ADCs is complex and because ADCs are comprised of multiple components, individual agent characteristics and patient variables can affect their disposition. To further improve the clinical use and rational development of these agents, it is imperative to comprehend the complex mechanisms employed by antibody-based agents in traversing numerous biological barriers and how agent/patient factors affect tumor delivery, toxicities, efficacy, and ultimately, biodistribution. This review provides an updated summary of factors known to affect the disposition of mAbs/ADCs in development and in clinical use, as well as how these factors should be considered in the selection and design of preclinical studies of ADC agents in development.

## 1. Introduction

The treatment of cancer and other conditions has observed significant progress and growth in treatment choices, with the exponential growth of carrier-based drug systems available. The inception of the use of monoclonal antibodies (mAb) to exploit the immune system and tumor-specific targeting presents an attractive solution to the small-molecule chemotherapy traditionally used in the treatment of cancer [1]. While immunogenic toxicities plagued early mAb therapies, current advancements in genetic engineering have allowed for the construction of fully human and humanized antibodies that are currently effective [2]. However, the most recent research into antibody-drug conjugates (ADCs), where highly potent cytotoxic drugs are conjugated to a mAb carrier, have grown into an active area of drug development, primarily in the treatment of malignancy. Now, having stemmed over four decades of intensive research, the challenges of immunotherapy agents are beginning to be translated into clinical practice, with numerous mAb agents and five ADCs currently on the market. There are ~85 ADCs in all phases of clinical trials and >75 early stage clinical trials for patients with solid tumors (Table 1) [3]. If we take into account non-conjugated mAbs, there have been >60 agents approved since the turn of the century (Table 2). 

Even though these agents have been in clinical use for nearly twenty-five years, the factors affecting the disposition of these antibody-based agents are still being discovered and evaluated. Identifying the causes behind pharmacokinetic (PK) and pharmacodynamic (PD) variability, especially bi-directional interactions with the immune system and the exposure–response relationship, as well as evaluating potential methods to individualize therapy, are essential to escalating their efficacy and thus clinical utility by limiting toxicities. Furthermore, major challenges remain in how to evaluate the safety and toxicity of these agents using preclinical and clinical models, as well as the variation observed within preclinical models. This high inter-patient variability is clinically important as ADCs have a narrow therapeutic index [1]. With the fast-paced research being performed in this field, this review includes new research and published information on factors that affect the PK and PD of mAb and ADC therapies. In addition, we provide additional insight into differences in preclinical models and how variations among models can affect their translation into human clinical trials. 

## 2. Pharmacokinetic Considerations

### 2.1. Pharmacokinetic Disposition

**Absorption:** Protein-based agents face numerous physiological barriers with oral administration (that have been well-characterized), necessitating the parenteral administration of these agents to attain systemic circulation [4]. Because of these limitations, antibody-based agents are required to be given either intravenously (iv) or subcutaneously (sc). In oncology, the most frequent route of administration is iv infusion, especially for ADCs, allowing for greater efficacy through the modulation of dissolution parameters to enhance the profile of cleavable-linker ADCs [5]. Ideally, ADCs are expected to have 100% bioavailability due to iv administration. In contrast, therapeutic mAbs utilized in treating a number of inflammatory conditions are able to be administered using sc injection, but with lowered bioavailability (between 50–80%) [6]. Despite the success of sc administration for mAb agents, this route of administration is unlikely for ADCs because of the potent cytotoxic drugs conjugated that can cause off-target toxicities mediated by immune cells in the skin. Subcutaneous administration may also pose an increased risk for patients with, or susceptible to, melanoma as expression levels of lysosomal-associated membrane protein 1 (LAMP1) may increase disease invasiveness [5]. 

**Distribution:** In general, mAb/ADC distribution is limited to the vascular and interstitial space due to their polarity and size [7]. Distribution of these agents typically occurs by a combination of three methods: convective transport, transcytosis across vascular epithelium, and passive diffusion [8]. Due to the size of mAbs and ADC agents, passive transport traditionally plays a minimal role in the overall distribution of these agents. The transport into tissues from the blood vessels by convective forces is slow and relies on pressure gradients [7]. Vascular transcytosis will rely on the structures of the blood vessel (membrane thickness, fenestration size) and the nearby tissue, which will all alter the degree of transport. For example, mAbs have a very low distribution rate into the brain due to the small fenestrations between cells of the blood vessels in the brain [7]. On the other hand, the leaky vasculature of tumors has larger gaps between cells, allowing for larger molecules to be transported into the tumors [7]. These are similar barriers that have restricted other carrier-mediated agents (CMAs; e.g., nanoparticles (NPs)) from entering tumors [9,10]. However, these barriers may be less restrictive to ADCs due to their smaller size (~10 nm) compared to NPs (~50 to 100 nm) [11]. 

Despite this potential size advantage, several additional factors within tumors can limit ADC distribution within tumors. For antibodies with higher affinities, distribution can be limited away from blood vessels if the target antigen is found on or near the vasculature. This phenomenon is known as the “binding site barrier” [12]. Lee and Tannock investigated this effect in a recent study of the distribution of cetuximab and trastuzumab on human epidermoid carcinoma (A431) and breast adenocarcinoma (MDA-MB-231) xenografts in murine models [12]. This study concluded that the distribution of antibodies was dependent on both dosage and time, and that hypoxic tumor regions exhibited worse antibody binding. As such, antibodies concentrated near the vasculature at early time points post-administration and displayed a heterogeneous distribution. With increasing dose or as time progressed, antibody distribution became more homogenous within non-hypoxic regions [12]. 

Monoclonal antibodies and ADCs experience the same barriers and distributive forces, but ADCs also bear a cytotoxic drug that can further affect ADC distribution. This is because the conjugated drug can additionally affect the binding affinity and internalization efficiency of an ADC. In addition, once the drug is released from the antibody carrier, the drug will carry its own unique distribution profile from the mAb/ADC. Specifically, small molecule drugs that can cross membranes, allowing them to dissociate beyond the target cell, can take advantage of the “bystander effect” and may be beneficial in tumors with heterogeneous target expression [13,14]. A study by Breij et al. revealed that murine patient-derived xenografts of solid tumors administered an ADC conjugated with monomethyl auristatin E (MMAE) experienced a complete response, despite the ADC target antigen only being expressed on 25–50% of cancer cells [15]. This strong reaction was alleged to be due to the bystander effect of MMAE [15]. Determining the best grouping of the antibody carrier and cytotoxic payload is therefore essential to both the safety and efficacy of ADCs. Through further characterization, we may be better able to tailor ADCs through the modulation of dose size, concentration, and binding affinity to prevent binding issues and further increase the efficacy. Through development of the next generation linkers and drugs, new ADCs may be able to increase the expression and potency of the bystander effect, thus reducing the need for proximity of the drug to the tumor and allowing for smaller molecules and/or more distant application [5]. The removal of the need for immediate proximity would allow for a greater effect despite occurrences of negative effects, such as a binding site barrier and poor drug delivery due to hypoxia.

Additional tumor factors could also play a part in the localized distribution of mAbs and ADCs, but require further study or identification of such factors. Characterizing these interactions between patient/tumor factors and antibody agents may help further enhance delivery to cancer cells and the overall clinical efficacy of these agents.

**Figure 1:**
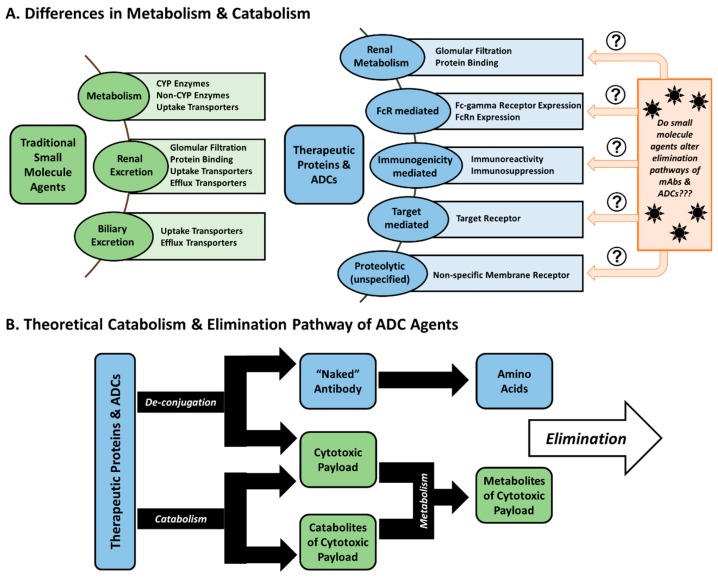
Metabolism and elimination differences of small molecules drugs compared to antibody-based agents. **(A)** Therapeutic proteins (including monoclonal antibodies and antibody-drug conjugates (ADCs)) undergo metabolism and catabolism in numerous different ways compared to traditional small molecule agents. **(B)** Ultimately, this results in ADCs relying on two separate, but concurrent, processes to eliminate both the antibody carrier and the cytotoxic drug.

**Metabolism and Elimination:** Compared to conventional small molecule drugs, the metabolism and elimination of antibody agents significantly differ. Small molecules characteristically undergo renal elimination or hepatic elimination, or are metabolized first to form metabolites which can more easily be eliminated (e.g., changes in molecular weight, changes in polarity) by these means. However, antibody-based agents are more complex, thus requiring a combination of specific and non-specific mechanisms to be eliminated (Figure 1). The catabolism of ADCs primarily takes place by non-specific macrophage and endothelial cell uptake and subsequent proteolysis in a number of tissues (such as the skin, muscle, and liver) [16]. These cells engulf and degrade antibodies through non-specific pinocytosis, where proteins are catabolized via lysosomal proteolysis [8].

Antibody agents, including both therapeutic mAbs and ADCs, can be eliminated by one of many specific mechanisms. The most commonly associated pathway is target-mediated clearance, which occurs when an antibody binds its target antigen on the surface of a cell before it is internalized and catabolized by the cell. However, this clearance mechanism is saturable, and can cause non-linear disposition, especially when agents are given at low doses and/or there is high expression of the target. These agents can also bind to Fc-gamma receptors (FcγR) expressed on cells of the mononuclear phagocyte system (MPS) and other immune cells, also leading to their internalization and catabolism. This method of elimination could be important for ADCs which target secreted/circulating proteins or those that develop immune complexes, as increased and rapid binding to FcγRs is observed with larger immune complexes [17]. Kasturirangan et al. recently published a report taking advantage of phagocytic pathways and MPS-based clearance as a means to neutralize and clear interlukin-6 (IL-6) with a bispecific antibody [18]. They observed that single antibodies and smaller immune complexes evaded FcγR-mediated clearance due to weaker FcγR interactions. In order to counter this effect, a bispecific antibody was constructed which targeted two unique epitopes of IL-6, therefore allowing the creation of larger, multi-branched immune complexes of IL-6. In vivo studies in C57BL/6 mice administered single-epitope binding mAbs and exogenous IL-6 verified the extended circulation of IL-6-mAb complexes in circulation. However, when mice were instead administered a bispecific antibody, rapid clearance of the IL-6 immune complex from circulation was observed, along with FcγR-dependent accumulation in the liver (a MPS organ) [18]. Such novel approaches illustrate the capability for improved drug design based on MPS interactions and understanding of how antibodies interact with these cells. 

### 2.2. Innate Immune System: Mononuclear Phagocyte System

As we strive to comprehend the unique pharmacology of antibody-based agents, it should be noted that apparent similarities exist between the altered disposition of NPs and antibody-based agents, and these mechanisms are ultimately believed to be driven by the MPS (also called antigen presenting cells; a part of the innate immune system). In fact, it appears that the MPS performs a significant role in the overall distribution and activation of both mAbs and NP drugs. As there is high inter-patient functional and phenotypic variability of the MPS, the varying characteristics of these agents lead to the observed variability in the disposition of these complex formulations [19,20]. Both types of agents are well-known to accumulate and distribute within MPS-rich organs (e.g., liver, lung, spleen) and coincidently are cleared via the same organs [21,22,23,24,25,26,27,28]. Likewise, the increased relative distribution of antibody agents to the liver, spleen, and lungs has been observed in both mouse and non-human primate models of cancer [27,29,30,31]. Finally, NPs and mAbs each display non-linear clearance (CL), most likely due to the saturation of MPS-mediated CL pathways [32,33]. A complete list, accompanied with functional and phenotypic differences among tissue-resident phagocytes, is reviewed elsewhere [34]. 

**Figure 2:**
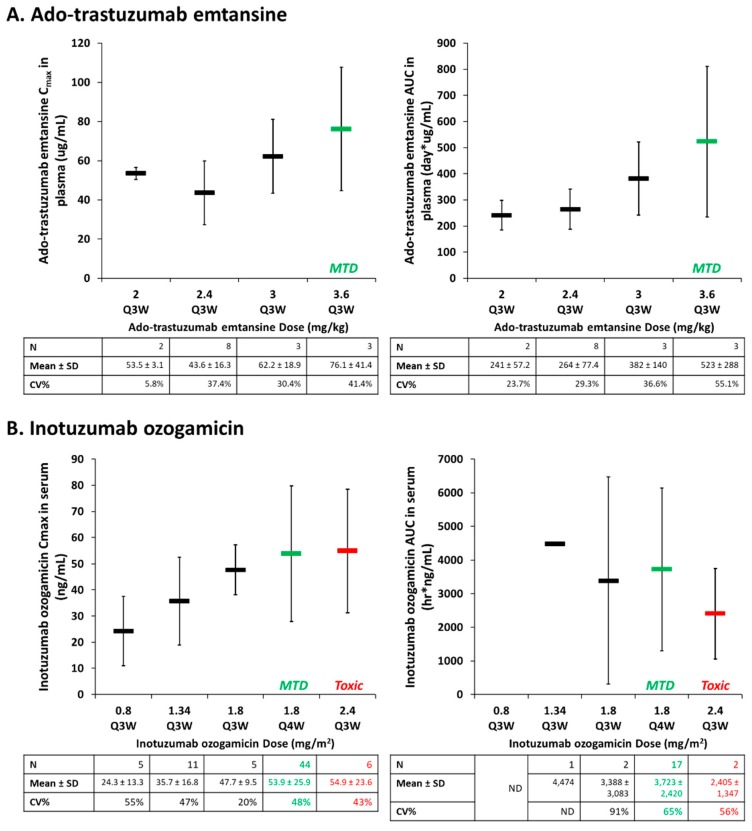
High variability in the pharmacokinetic (PK) of antibody-drug conjugates (ADCs) as represented by the relationship between ado-trastuzumab emtansine **(A)** and Inotuzumab ozogamicin **(B)** dose and PK parameters (Cmax, area under the curve (AUC)) in serum. Mean ± standard deviation (SD) of patients for each treatment group are represented by the rectangular bar. There was high inter-patient pharmacokinetic (PK) variability in studies of both solid (breast cancer; ado-trastuzumab emtansine) and hematologic (B-cell lymphoma; inotuzumab ozogamicin) malignancies. The high PK variability observed in these ADC agents may be related to the variability in the mononuclear phagocyte system (MPS). CV%, coefficient of variation; Q3W, every three weeks; Q4W, every four weeks.

Similar to antibodies and ADCs, NPs also present high inter-patient PK and PD variability. A prior meta-analysis that compared the inter-patient variability of small molecule agents and corresponding liposomal formulations in patients demonstrated a significant increase in plasma area under the curve (AUC) exposure variability for liposomes (66%) versus small molecules (31%) [35]. In comparison, the inter-patient CL variability in patients administered trastuzumab has been reported to be 43% [36]. ADCs appear to demonstrate similar patterns of high inter-patient variability as well, which complicates understanding the dose–response relationship for these agents. The full PK profile of ado-trastuzumab emtansine (including ADC, total antibody, and DM1 concentrations) was determined when administered together with paclitaxel in a phase Ib/IIa study of HER2-positive metastatic breast cancer patients [37]. The association between ado-trastuzumab emtansine dose and PK disposition (Cmax and terminal AUC) in serum is shown in Figure 2A. There was high inter-patient PK variability in ado-trastuzumab emtansine AUC and Cmax in plasma across multiple doses, with increasing variability as doses approached the maximum tolerable dose (MTD). In a recently FDA-approved ADC in 2017, inotuzumab ozogamicin, used in the treatment of hematologic malignancies, was evaluated as part of a phase I study in patients with B-cell non-Hodgkin’s lymphoma that progressed after two prior therapies [38]. Like ado-trastuzumab emtansine, PK parameters in serum of inotuzumab ozogamicin demonstrate similar high inter-patient variability (Figure 2B). For both agents, the concentration verses time profiles of the ADC and total antibody (ADC plus cleaved free antibody) in serum were similar. The PK variability of the released cytotoxic drug (DM4 and ozogamicin, respectively) was also similar to the PK variability of the ADC. Furthermore, serum exposures overlapped at doses leading to the MTD in both agents (Figure 2). Interestingly, similar PK variability exists in both a solid tumor (breast cancer) and hematologic tumor (B-cell lymphoma) patient population, suggesting that common traits between these pathologically different malignancies may be responsible. As a relationship between PK variability with traditional covariates or anti-drug antibody (ADA) titers was not found in these studies, the high inter-patient variability of ADCs may be related, with similar variability seen in MPS function.

Both an agent’s and a patient’s physical characteristics can affect the disposition of mAbs and ADCs. ADCs demonstrate a faster CL when the mAb carrier is linked with a greater number of ligands. This is exemplified by ADCs like ado-trastuzumab emtansine which, when compared to non-conjugated trastuzumab alone, is cleared more rapidly (3- to 4-fold) in both mice and humans when administered at similar doses [39,40,41]. This is ascribed to the ability of MPS cells to identify and internalize more hydrophobic, “non-self” agents. Furthermore, a patient’s body habitus (i.e., body weight) has previously been observed to affect the pharmacology of both NP and antibody agents. Population PK studies of trastuzumab and ado-trastuzumab emtansine in patients with HER2+ metastatic breast cancer and brentuximab vedotin in CD30-expressing hematologic malignancies have shown that increased body weight correlates with increased CL [36,42,43,44]. This is consistent with the altered MPS function for patients with a larger body mass and weight due to the increased MPS function reported in an obese population [19]. Finally, tumor burden has also been shown to be a relevant covariate related to CL, where patients with an increased number of metastatic sites had increased CL [45,46]. The PK of MVT-5873 was evaluated in non-tumor bearing mice and mice bearing BxPC3 human pancreatic cancer xenograft models [47]. In non-tumor bearing mice, serial trough serum concentrations were relatively constant, where in contrast, there was a consistent decline in serum concentrations in tumor-bearing mice. There was also an inverse relationship between tumor growth curves and serum exposures of MVT-5873, suggesting that the presence of tumors and tumor burden are associated with enhanced clearance of MVT-5873. This enhanced clearance has been seen with other mAb agents, such as trastuzumab, where tumor burden was a significant covariate for CL highlighted in its population PK analysis [36]. 

Ado-trastuzumab emtansine and brentuximab vedotin are two ADCs that target antigens preferentially on the surface of cancer cells to provide clinical efficacy with a manageable safety profile [48,49,50]. While both of these agents have an average DAR of 3.5–4, brentuximab vedotin and ado-trastuzumab emtansine differ in the ability of their linker to be cleaved [51]. However, while ado-trastuzumab emtansine has not been shown to demonstrate accumulation due to its non-specific distribution in highly perfused organs, brentuximab vedotin shows some accumulation in the liver [52,53,54]. These data imply that specific ADC characteristics could make brentuximab vedotin more easily recognized by the immune system than non-conjugated (i.e., ‘naked’) mAbs. In fact, immune checkpoint inhibitor-associated immune-mediated hepatitis (IMH) is an evolving issue within the field, where hepatotoxicity is found to range between mild to severe to life- threatening [25,55]. All six of the mAb agents targeting immune checkpoints (ipilimumab = CTLA-4; pembrolizumab and nivolumab = PD-1; atezolizumab, avelumab and durvalumab = PD-L1) have demonstrated some degree of liver accumulation, complicating the duration of responses and survival benefit resulting from these agents [25,55,56,57,58]. In addition, a combination of agents resulted in an increased incidence of hepatic toxicities, as demonstrated in patients with melanoma receiving a combination of ipilimumab and nivolumab [59,60,61]. However, it has not been determined if this hepatic accumulation is due to certain mAb or ADC characteristics (such as linker choice) or pharmacologic exposure levels of these agents. The development of reliable probes to predict patients at increased susceptibility to liver injury would be beneficial and help to optimize precision dosing decisions for individual patients, especially when used in combination.

## 3. Physical Characteristics of mAbs & ADCS

Several recent reviews describe how the composition of ADCs (such as target selection, linker choice, and choice of cytotoxic drug) affects their disposition, and as such are not covered in this review [20,62]. However, the physical characteristics and chemical alteration/degradation of mAbs/ADCs are also contributing factors that must be accounted for in the design and selection of more reliable and stable agents. Additionally, the PK of antibody-based agents has improved their delivery as innovations are continuously being implemented in the field.

### 3.1. Size

The size of therapeutic proteins varies and this difference in size can affect the disposition in numerous ways. Full IgG mAbs (~150 kDa), Fab fragments (Fabs; ~50–66 kDa), serum albumin (~66 kDa), and streptavidin (~53 kDa) are examples that have been examined. These proteins were individually evaluated in murine HeLa tumor models and protein concentration in blood was measured over time [63]. The slowest clearance was observed by full IgGs when compared against streptavidin and serum albumin and Fabs demonstrated the highest clearance; these results were found to be directly related to the molecular weight of each agent. Compared to full IgG antibodies, Fab fragments are less than one-third the size, but clearance differences were found not to be proportional to size, as Fabs demonstrated a 10-fold greater CL (1.1 mL/h vs. 0.1 mL/h), while the Vd remained similar among all agents (~1.5 mL). The CL of Fabs is faster because these molecules are able to undergo more traditional mechanisms of elimination than IgGs, such as renal elimination or hepatic excretion versus endocytosis. In terms of pharmacological benefit, full IgG mAbs or ADCs (i.e., larger proteins) have an increased circulation time in the body, providing the opportunity for less frequent dosing intervals and increased exposures over time, but also potentially increase the risk of off-target effects and toxicities.

While only one ADC has been approved for use in solid tumors and numerous others have been discontinued after clinical trials, there is a growing general consensus that smaller antibodies (or fragments) may provide more efficient penetration into tumors than full sized antibodies [64,65,66,67,68]. While smaller ADCs may provide improved tumor penetration, due to their rapid clearance and disposition, retained potency and efficacy compared to their whole antibody counterparts requires additional evaluation. Therefore, half-life differences of mAb fragments compared to traditional mAbs should also be considered in dosing strategies, as smaller mAb fragments may provide greater tumor penetration but may not be held in circulation long enough to efficiently provide enough agent to the tumor. In addition, due to the decreased size of the biologic component, the properties of the conjugated cytotoxic drug will be more important and will affect the ADC profile more than ‘whole antibody’ ADCs. Without additional data from pre-clinical studies and clinical efficacy/tolerability studies, it is still too early to determine which small-antibody format(s) or conformation(s) are the most promising. 

### 3.2. Drug-Antibody Ratio (Dar)

The number of small molecule cytotoxic agents conjugated to a single antibody, or the drug-to-antibody ratio (DAR), is essential in establishing the efficacy of an ADC. While the optimal DAR to attain maximum efficacy has not yet been determined, this trait is likely to be highly dependent on other ADC variables and not the cytotoxic drug selected. Additionally, production techniques result in variable DARs within a single solution; but managing heterogeneity is complicated by the number of potential conjugation sites on the surface of an antibody, such as lysine and cysteine residues [3]. This poses several challenges in the characterization and optimization of ADCs. An ADC may not provide a significant enough anti-tumor response when too few molecules are attached. If too many molecules are attached, the ADC might become unstable, display increased toxicity, and produce varied PK and PD properties [69]. In addition, high DAR ADCs result in increased aggregation due to the added hydrophobicity of the conjugated cytotoxic drugs [69,70,71]. 

There have been extensive studies on the effects of DAR on ADC properties, which have also been reviewed [20,72,73,74]. 

**Figure 3:**
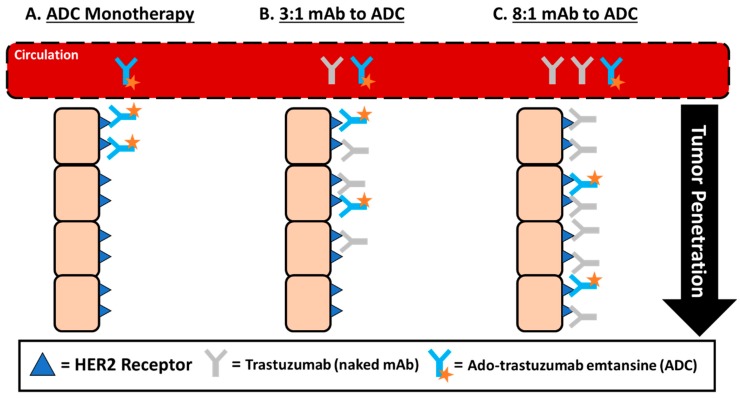
Improved ADC tumor penetration by administering ADC in combination with naked antibody carrier. (**A**) The tumor distribution of ado-trastuzumab emtansine monotherapy administered at 3.6 mg/kg results in only perivascular tumor distribution due to rapid binding after being transported into the tissue from systemic circulation. (**B**,**C**) Improvement in the tumor penetration of ado-trastuzumab when co-administered with trastuzumab at a dose sub-saturating or saturating dose of trastuzumab. In theory, these results are due to trastuzumab competing for binding sites, requiring ado-trastuzumab emtansine to penetrate further into tumors to find available binding sites (tumor specific effect) or due to trastuzumab reducing the uptake of ado-trastuzumab emtansine by the mononuclear phagocyte system (MPS) and increasing the serum exposure, which is associated with the greater tumor delivery that has been reported for nanoparticles (NP) agents (reduced systemic clearance effect).

An alternative strategy that has been recently proposed is the use of high DAR ADCs administered in combination with naked antibody counterparts. Cilliers et al. sought to understand the association between DAR and tumor penetration and intra-tumoral distribution in a murine breast cancer xenograft model [75]. In this study, a mixture of trastuzumab (naked antibody) and ado-trastuzumab emtansine (ADC) in defined ratios (3:1 and 8:1) was utilized to artificially reduce the effective DAR based on a constant antibody concentration administered. Surprisingly, both administered ratios of mAb to ADC were shown to improve ADC tumor penetration (Figure 3), which translated into a clinically significant two-fold increase in median survival when compared to ADC monotherapy. While this method decreased the potential amount of cytotoxic drug able to be delivered to each cell, the number of cells internalizing ADC (and thus cytotoxic agent) was also increased. This result is counterintuitive to what is typically expected, but by competing for binding sites, the ADC penetrated further into the tumor to find available binding sites, thus improving the effectiveness of ADC therapy. This mechanism could be beneficial and unique for utilizing high DAR agents, so long as the naked antibody carrier is also available.

Furthermore, the conjugation site plays a distinct role in altering ADC pharmacology. To demonstrate this effect, Strop et al. generated solutions of ADCs with a mean DAR of 1.7, but altered the site of conjugation on the antibody backbone to precise points using a novel enzymatic technique [76]. This study demonstrated how the conjugation site influenced the stability of the linker, where light chain conjugation was cleaved faster than if conjugated to the heavy chain in mice; but the linkers were stable in rats. Moreover, conjugation to the heavy chain resulted in dramatically altered PK and faster CL of ADCs compared to either non-conjugated mAbs (i.e., naked antibodies) or light chain conjugated ADCs in rats. More recently, engineered ADC technologies have been applied to reduce the heterogeneity of the DAR within a given compound. These modifications traditionally revolve around the partial reduction of cysteine residues to guide conjugation opposed to lysine residues, which are found in great numbers on antibodies but can lead to non-specific conjugation [77,78,79,80]. The uses of site-specific conjugation technologies can allow for improved studies on linker position and cytotoxic drug differences in order to optimize and direct the pharmacology of ADCs to improve the therapeutic index of these agents. As literature around this subject is broad and constantly evolving, additional articles exist with additional in-depth insights [81,82,83,84].

### 3.3. Surface Engineering & Chemical Alterations

A common practice to mediate the disadvantages of certain agents is to modify how the body handles drug carriers by changing the structure. The most frequent modifications to therapeutic proteins are by glycosylation or PEGylation. However, it should be noted that increased structural modification to the ‘native’ IgG structure can also increase the risk of non-specific binding of these proteins [85]. In addition, chemical modifications, such as oxidation, can drastically affect the disposition of proteins. 

**Glycosylation**: Post-translational modification of proteins occurs naturally within the body, and most commonly by the conjugation of carbohydrates (i.e., glycans) to the side chain of exposed amino acids—a process known as glycosylation. This modification occurs naturally due to the use of eukaryotic cell lines to produce therapeutic proteins, though several factors (such as the culturing conditions and selection of cell line) influence the site and extent of glycosylation [86]. As such, the process of glycosylation is a complex post-translational modification that can influence the biological activity, protein structure, formulation characteristics (e.g., solubility, antigenicity, stability), and ultimately, its PK [86,87]. However, the location and the amount/type of glycosylation radically affect protein disposition, such as modulating effector functions, receptor binding, and signaling (including antibody-dependent cellular cytotoxicity (ADCC) and complement-dependent cytotoxicity (CDC)) [87,88]. Additionally, N-linked glycosylation on smaller proteins, such as diabodies, has been shown to increase systemic exposure [89]. While numerous conflicting studies exist that argue if glycosylation of the Fc region affects serum clearance, it is generally accepted that higher levels of mannose-5 glycan forms are cleared more rapidly [90,91]. Ado-trastuzumab emtansine was actually glycol-engineered to allow for payload addition to avoid interference caused by drug-loading and alleviating the need to remodel the antibody carrier [92]. Furthermore, these data provide justification for Fc glycosylation engineering as a rational strategy to improve the PK (increase exposure) and PD (safety and efficacy) of antibody agents. However, due to the differing levels of glycosylation and glycoforms present in current manufacturing methods, the analysis of heterogeneous populations requires a thorough analysis of different drugs (i.e., glycoforms) within a given solution and its change across time. The authors refer you to several reviews for more detailed information on the effects of glycosylation on therapeutic antibodies [93,94,95]. 

**PEGylation**: The addition of the theoretical non-immunogenic poly-ethylene glycol (PEG) polymer chains is an additional method of antibody modification to overcome certain disadvantages. Traditionally, PEGylation improves agent characteristics, such as providing better solubility and prolonging circulation in the body [96,97,98]. This happens as the addition of PEG defends against degradation by enzymes, slows filtration by the kidneys due to the increased size, and evades detection by MPS cells [99]. However, as PEGylation adds a steric limitation or surface charge modification to the agent it has conjugated, it can then affect binding affinity to its target. Thus, control of the site of conjugation and conjugation to individual sites should be evaluated. To date, PEGylation on ADCs has mainly served as a linker to both improve solubility and reduce the formation of aggregates, as well as to serve as a conjugation point for the cytotoxic drug. Burke et al. studied the effect of varying the lengths of PEG side chains to determine the alteration in PK parameters [100]. Sprague-Dawley rats were administered a dose of 3 mg/kg MMAE (8:1 MMAE:mAb) with varying lengths of linking PEG-chain (2 to 24 PEG-block polymers) and the amount of antibody was monitored over time. The result was that slower clearance was observed with increasing length of the PEG-chain (from PEG_2_ and PEG_4_), and only minor differences in PK parameters were observed from PEG_8_–PEG_24_. However, a disadvantage of such large modifications is that these PEG molecules are not efficiently eliminated, remaining in circulation even after the protein/antibody has been catabolized, potentially complicating therapies that require prolonged treatment durations due to long-term PEG retention and effects within the body [101,102]. In addition, using PEG on Fab fragments increases their time in circulation, and could serve as an advantage for prolonging the circulation of these rapidly cleared molecules [97]. However, the addition of PEG to antibodies to reduce the clearance by the MPS may also result in increased PK and PD variability of these agents, as has been seen when comparing the PK variability of PEGylated (higher inter-patient PK variability) and non-PEGylated liposomes [35].

**Oxidation:** Oxidation is a common chemical modification that can occur during the production of therapeutic proteins in culture or storage due to changes in pH or prolonged exposure to light [103,104]. While multiple amino acids can be affected by this reaction, the four methionine residues located in the Fc domain carry special importance, as oxidation of these residues has been linked to reduced antibody stability and altered Fc-regulated effector functions, and binding affinity to various substrates [103,104,105]. Oxidation of tryptophan residues can also be observed and has been linked to loss of stability and loss of affinity [104]. A recent publication demonstrated that the oxidation of Met252 of the human IgG1 Fc region resulted in a significant decrease in half-life (>4-fold reduction) and was related to decreased binding to the Fc neonatal receptor (FcRn) [106]. Moreover, the oxidation of Trp residues within the Fab region of human IgG1 antibodies resulted in a loss of antibody potency, or complete loss of binding and effector function [104,107]. 

### 3.4. Charge and pH Engineering

The net charge a protein carries is a crucial variable influencing its non-specific interactions due to electrostatic interactions while within circulation and tissues. Charge is determined by the pH, where the antibody carries no net electrical charge, referred to as the isoelectric point (pI) [108]. However, just as DAR is heterogeneous in ADC production, ADC solutions carry heterogeneous charge states (relative to pI properties and surface charge) due to current manufacturing and isolation techniques. Cationization of antibodies, where the pI is raised/more basic, typically results in improved binding to anionic sites on the cell surface, resulting in higher tissue accumulation and increased systemic CL [108]. This means that antibodies with a higher pI have faster systemic clearance and lower bioavailability due to these increased non-specific interactions. Shifting the pI by one unit can produce measurable changes in the kinetics and distribution within tissues [109,110]. One study measured the effect of altering an antibody’s pI by 2 units (from pI 7 to 9) and an increase in the plasma CL by 28-fold [111]. However, minor alterations to the pI (<1.0 unit) do not develop altered PK profiles, suggesting that these minor differences may not warrant additional PK considerations [108,112]. In addition, antibody cationization has also been exploited to encourage receptor-mediated endocytosis and extravasation into tissues due to increased electrostatic interactions (i.e., positively charged antibody and negatively charged cell membranes) [108,113,114]. Thus, it is critical to characterize isolated charge variants to evaluate individual differences in PK disposition, as overall ADC PK variability can be due to heterogeneous solutions of charge variants. 

## 4. Host-Associated Factors and Disease Status

Host-associated factors, such as gender and organ function, have been associated with the altered disposition and increased toxicity of other advanced formulations, such as NP and other CMAs [19,20]. While these differences are thought to be linked to immune cell activity or mediators/regulators of phagocytic immune cells (e.g., monocytes, dendritic cells, MDSCs), additional studies are required to demonstrate the strength of these relationships. 

### 4.1. Presence of Liver Metastases

Patients with different types of tumors in different locations (e.g., liver) can present with altered immune status, including the alteration of MPS function, along with different PK dispositions of CMAs, which presents a unique problem in determining the optimal dosing regimen for an agent. A prior phase I PK study of NP S-CKD-602 observed that liver tumors were a significant covariate for increased CL within PK models [46]. In addition, the S-CKD602 inter-patient PK variability was able to be explained by the presence of metastatic liver tumors. This was determined as patients with liver tumors had a Cmax ~1.5-fold higher than patients without liver tumors. Together, these data suggest that patients with liver metastases are at increased risk for a lower response to S-CKD602 therapy. This result was unique as most studies demonstrate decreased rates of CL of small molecule drugs in patients with liver tumors [115,116].

In a recent evaluation of patient data compiled from previous phase I studies utilizing pembrolizumab in either non-small cell lung cancer (NSCLC) or melanoma (Keynote 001, 002, and 006 studies), investigators sought to determine if liver metastases were associated with differences in response [117]. Their analysis found that the presence of liver metastases in patients with melanoma was associated with both reduced response to therapy and decreased (shortened) progression-free survival (PFS) compared to those without metastases (ORR, 30.6% vs. 56.3%; median PFS, 5.1 vs. 20.1 months; *p* < 0.0001). Similar results were found in patients with NSCLC (1.8 vs. 5.1 months; *p* = 0.0094). This is the first study to show that the presence of liver metastases was associated with reduced response and PFS with treatment of an immunotherapy. Similar to the prior study in NPs, increased/altered immune cell function could be responsible for this outcome, providing a mechanism for these outcomes. 

### 4.2. Sex and Body Habitus

Despite the focus placed on precision medicine, differences in sex are commonly neglected in everyday clinical practice. Case and point, most preclinical studies are first evaluated in male animals due to the belief that hormonal variations in female animals leads to inherent variability compared to males [118]. Despite the NIH’s guidance document released in 2015 on “sex as a biological variable” (SABV), such policies do not require researchers to power studies or implement study designs to ensure study comparing the sexes [119,120]. Only one study exists with a focus on evaluating the impact of gender on an ADC–gemtuzumab ozogamicin [121]. This data came from 58 patients (29 men, 29 women) enrolled in a phase II study to determine the safety and efficacy of gemtuzumab ozogamicin in adults with acute myeloid leukemia (AML). Overall, the study concluded that there was no difference in the PK of the ADC, naked antibody, or unbound drug based on gender or age. However, due to significant PK variability (e.g., 0.254 ± 0.229 (CV% 90.1%) and 0.277 ± 0.232 (CV% 83.7%) L/h in men and women, respectively), the differences associated with age could be masked due to other covariates, such as the interaction with the MPS, and thus warrant further analysis. While other studies of mAb-based agents have investigated gender differences during post-hoc analyses, the associations have not been strong enough to warrant a change in their method of administration, but these results may be due to methodological and statistical limitations.

Historically, it is controversial whether the disposition of antibody-based agents is affected by body weight or composition, though evidence does exist [43,122,123]. A population analysis of five phase I to III studies (totaling 671 patients) administering ado-trastuzumab emtansine found that body weight, serum albumin, and tumor burden demonstrated significant effects on its disposition [123]. Of these covariates, the greatest effect on disposition was seen with body weight, where patients with a higher body mass displayed both higher CL and Vc [123,124]. Body weight was also shown to affect Cmax concentrations and steady-state AUC exposure of ado-trastuzumab emtansine. Body weight and BSA demonstrated similar effects in a population analysis (314 patients across five trials) of brentuximab vedotin trials [43]. In the case of both ADCs, a higher CL and lower AUC exposure were observed in patients with larger body habitus. While these studies support the decision to employ weight-based dosing strategies, significant PK variability still exists among patients despite the use of this weight-based dose normalization and is consistent with the higher CL of mAbs and ADCs also being associated with the higher function of MPS cells viewed in overweight (body mass index (BMI) > 25) patients [19,20]. 

### 4.3. Biochemical Mediators of Immunity in Blood

Standard population PK analyses routinely evaluate patient covariates to extrapolate inter-patient PK variability and numerous biochemical factors circulating in the blood have been suggested to regulate immunity and tissue effectors. Hormones and chemokines are small, biochemical signaling molecules released into systemic circulation before traveling to distant tissues to control and regulate various bodily functions. It appears that they, directly and indirectly, affect the MPS, subsequently altering the PK and PD of CMAs, including ADCs [125,126,127,128,129]. Sex hormones have been demonstrated in multiple studies to have in vitro regulatory effects on macrophages and lymphocytes, including FcγR expression [130,131,132,133,134,135,136]. Of particular interest are the reports of estrogen and its other various forms promoting MPS cell phagocytic activity in vitro. In a study by Hu et al., Lewis rats (both male and female) were administered exogenous 17β-estradiol [137]. After exogenous estradiol administration, the secretion of interleukin-1 (IL-1) by macrophages was two-fold greater in rats that received estradiol than those that did not (41,632 vs. 21,125 counts/min/well, respectively). The most potent form of vitamin D3, calcitriol, also appears to be an important mediator of FcγR expression, as well as an alternative regulator of key immune cytokines in circulating phagocytes (IL-1, IL-6, TNFa) [138,139,140,141,142,143]. There is growing evidence to indicate that both chemokines and their receptors control the activation and translocation of MPS cells, such as monocyte/macrophage differentiation within tissues, which ultimately affects the PK of complex carriers. For example, CCL2 and CCL5 have previously been reported to be essential for the migration of monocytes from systemic circulation and alterations in this chemical signaling within tumors produce a local or systemic PK effect of CMAs [144,145,146,147]. 

The effects of these mediators might be more pronounced within tissues, as activated macrophages and other immune cells can be influenced by regional mediators (e.g., calcitriol and cytokines) if they reach pharmaceutically-relevant concentrations. However, these mediators have yet to be extensively evaluated in clinical trials or in in vivo experiments of preclinical animal models to directly quantify the effect of these hormone/chemokine mediators on PK/PD. 

### 4.4. Renal or Hepatic Impairment

According to guidance documents drafted by the FDA, the evaluation of mAb agents in special populations (e.g., renal and hepatic impairment) is not needed [148,149]. The reasoning is that renal and hepatic impairment is not expected to change the disposition of these agents because their clearance and disposition is not as affected by the kidneys and liver. Moreover, several case reports have reported that the PK of bevacizumab, cetuximab, rituximab, or trastuzumab was not affected in patients undergoing hemodialysis [150,151,152]. While the majority of antibody-based agents may not be influenced, certain agents able to pass glomerular filtration (<60 kDa) could be eliminated in part by the kidneys [153]. These smaller derivatives (such as Fab fragments) can pass the cutoff, displaying a gradual decrease in CL, and thus increased accumulation/exposure, in patients with kidney disease. This is supported by the results of Czock et al., who evaluated patients diagnosed with severe renal failure or end stage renal disease (ESRD) and reported upwards of a three-fold CL reduction of smaller proteins (<50 kDa) in this population [154]. 

In addition, the kidneys may serve as an elimination pathway for therapeutic proteins via internalization and catabolism (i.e., phagocytosis, pinocytosis, fluid transport mechanisms) versus filtration for smaller therapeutic agents [8,155,156,157]. A prior study used a mouse model to track the differences in the distribution and elimination of a MOPC-21-targeted whole antibody, F(ab)_2_ fragments, and Fab fragments [155]. This target is particularly useful in studying antibody metabolism in non-tumor tissues as it has no known binding sites within the body. Overall, plasma and MPS-related organs (lung, liver, spleen, gut) had the highest levels of exposure to whole antibody and lowest levels in the kidney. The greatest percentage of whole antibody catabolism was found to be 72% in the gut, 20.5% in the liver, and 3.6% in the spleen. However, Fab fragments are cleared ~35 times faster compared to whole antibody and found to be primarily catabolized in the kidney (73.4%), gut (22.9%), and spleen (3.1%). More recently, the glycosylation products conjugated to a mAb have also been linked to non-specific (i.e., non-receptor mediated) catabolism occurring in organs with a rich blood supply and endothelial cells, such as the muscles, skin, and gastrointestinal tract [158]. These data suggest that liver impairment could present a minor effect on whole antibody elimination, and kidney impairment can have a significant effect on Fab fragments. These studies also highlight how functional studies compared to receptor analysis may provide significant insight to address and understand the inter-patient distribution. 

### 4.5. Neonatal Fc Receptor (FcRn)

The prolonged serum half-life of antibodies in circulation due to their interaction with FcRn has been well-characterized [159,160]. In general, it is believed that circulating IgGs must first be internalized by either fluid-phase pinocytosis or non-specific endocytosis before they can interact with FcRn receptors and to be recycled/expelled out of the cell [161]. However, FcRn is widely expressed throughout the body and has been suggested to have differing functions based on the residing tissue. Chen et al. examined the effect of FcRn expression on IgG1 biodistribution using wild type (WT) and FcRn-knockout (KO) mice [162]. KO mice demonstrated a decreased tissue to blood exposure ratio compared to WT mice within muscle, fat, and skin; alternatively, this ratio was increased in the kidneys, liver, and spleen within KO mice. The differing effects observed within each tissue were explained by the different functions of FcRn within each tissue. For example, FcRn may serve mainly to transport antibodies from tissue-to-circulation in the liver and spleen, while in muscle and skin, the opposite movement from circulation-to-tissue occurs to deliver antibodies into these tissues [162]. Recently, ADCs have also shown efficacy in glioblastomas, suggesting that ADCs are able to cross the blood-brain barrier (BBB) [163]. This result is unexpected as the BBB restricts plasma proteins from crossing due to the tight junctions of endothelial cells, so further studies are necessary to determine the mechanisms of transport. This is further confounded as FcRn-mediated transport appears to transport proteins only in the brain-to-blood direction [164,165,166]. Therefore, it is also suggested that this transport may only occur at the tumor-BBB interface and not normal BBB. 

Several reviews have been published on how to further improve the half-life of antibody-based agents by optimizing their interaction with FcRn and therefore altering their intracellular transport [167,168,169,170]. By modulating the FcRn–IgG interaction in an attempt to alter PK parameters, numerous investigators have either extended (improving efficacy and reducing dosing frequency) or shortened (to control known toxicities or for diagnostic evaluation) an agent’s half-life [167,168,169,170,171,172,173,174,175,176,177]. However, ADC-FcRn associations are still under investigation as varying DARs of brentuximab vedotin have demonstrated differences in FcRn affinity: a DAR of 2–4 decreased FcRn affinity, while a DAR of 8 increased FcRn affinity compared to an unconjugated antibody [178]. Thus, descriptive research into both the number and location of the cytotoxic drug’s effects on half-life are still needed and further points out the necessity for the homogenous preparations of ADCs. 

### 4.6. Fc-Gamma Receptors (FcγR)

FcγR expression in various tissues and circulating immune cells is another element to analyze in the thorough evaluation of antibody-based agents. These receptors are critical as cells of the MPS naturally serve as a clearance mechanism for endogenous antibodies and immune complexes [179,180,181]. In addition, myeloid cells and lymphocytes express a number of FcγR isoforms that interact with circulating monomeric or aggregated IgGs and opsonized substances [179]. However, these receptors carry differing affinities, depending on the arrangement of IgG [182]. For example, CD64 (FcγRI) is the only Fc-receptor that can bind monomeric IgGs with high affinity (~10^7^), while other receptors, such as CD32 (FcγRII) and CD16 (FcγRIII), primarily bind aggregated IgGs at a lower affinity (~10^6^ to ~10^4^; roughly 10 to 1,000x less efficient to CD64) [179,182,183,184]. Because of the variation in affinities and types of these FcγRs, varying expression profiles can result in significant changes in the effector functions of immune cells, especially phagocytic MPS cell’s ability to clear antibodies from circulation-ultimately affecting their PK and PD disposition.

Previous reports by Abuqayyas et al. stated that FcγR expression did not affect the PK of mAbs [185,186]. In both studies, an IgG1 agent was administered at levels ranging from 0.04–0.4 mg/kg and its PK evaluated in WT mice and KO mice strains (a FcγRI/RIII KO and a FcγRIIb KO) [185,186]. IgG1 plasma clearances were similar within all mouse strains and at all doses. However, the doses utilized in these studies were 100-fold and 250-fold lower than “therapeutic doses” of pertuzumab (30 mg/kg) and trastuzumab (100 mg/kg), respectively, required in murine models [185]. As a result, the lack of effect observed has a high likelihood to be due to the “micro-doses” of IgG1 agents in this study. Despite this dosing difference, higher exposures of the IgG1 agent were observed in the liver (an MPS organ) in the KO mice [185]. Furthermore, several studies have also reported that difference in MPS function and FcγR expression between mice and humans is different [125,179,182,187,188,189]. Furthermore, the primary dose-limiting toxicities to ADCs (e.g., thrombocytopenia) appear to be due to FcγR-driven cytotoxicity on FcγR-bearing immune cells [190,191]. Thus, these preclinical evaluations do not definitively demonstrate that FcγRs on MPS cells do not affect mAb/ADC PK, but additional studies at clinically-relevant doses are needed. 

In spite of these data, recent studies suggest that the FcγRs on tumor-associated macrophages (TAMs) modulate the TAM-ADC interaction, regardless of the antigen-binding moiety. In murine models bearing CD30-positive L-428 xenografts (a Hodgkin lymphoma model), both anti-CD30-vcMMAE and a non-binding replicate (IgG-vcMMAE) provided similar anti-tumor activity, resulting from similar drug release within tumors [192]. The L-428 tumors, along with five additional murine xenografts of hematologic malignancies, were also confirmed by immunohistochemistry (IHC) to correlate TAM infiltration with the anti-tumor activity of the non-binding IgG-vcMMAE ADC. Upon mutating the Fc-region of this non-binding ADC to ablate FcγR binding, anti-tumor activity was lost in a majority of these high-TAM infiltrating xenograft models. However, additional correlative studies within clinical trials are still needed to determine if this effect persists outside of these preclinical models. In addition, engineering the Fc-region of an ADC to mediate its interaction with FcγRs may serve as a viable method to optimize the PK and PD profile. 

## 5. Pharmacologic-Associated Factors

### 5.1. Drug–Drug Interactions (DDIs)

Small molecule agents traditionally pose a minimal risk of DDIs with mAbs/ADCs, as these agents traditionally utilize different elimination pathways, unless the drug has an effect that alters the function of cells involved in the clearance of mAbs/ADCs (either due to a drug’s mechanism of action or cytotoxicity) (Figure 1). Furthermore, it is also possible for the small molecule cytotoxic drugs to affect the overall immunogenicity of protein-drug conjugates [193]. 

Pertuzumab (Perjeta^®^; Genentech Inc., South San Francisco, CA, USA) is a humanized anti-HER2 mAb agent with a distinct mechanism of action from trastuzumab. As pertuzumab was initially intended to be co-administered with other agents, potential DDIs were evaluated in phase I studies, where the PK of pertuzumab (alone and in combination with trastuzumab and docetaxel) was evaluated in patients with metastatic breast cancer [194,195]. The mean serum Cmin after the administration of pertuzumab was 35.0 µg/mL when administered as monotherapy or 63.6 µg/mL when in combination with trastuzumab and docetaxel. This ~two-fold reduction in clearance, and corresponding increase in trough exposure, hints that altered PK and PD results from combining mAb agents when the target antigen is the same (i.e., HER2).

Dalotuzumab is a humanized IgG1 antibody targeting the insulin-like growth factor receptor type 1 (IGF-1R) receptor and has primarily been studied in the treatment of solid tumors [196,197]. This agent has been studied when co-administered with either a small molecule chemotherapeutic agent or an anti-EGFR antibody due to a suggested synergistic effect on tumor growth inhibition [196,197,198]. The phase I studies of dalotuzumab administered as a single agent were well-tolerated by patients with advanced solid tumors [199]. More recently, a study of a triple combination of dalotuzumab, cetuximab, and irinotecan was performed in Japanese patients with metastatic colorectal cancer [197]. Doi et al. reported that the co-administration of cetuximab and irinotecan with dalotuzumab increased its AUC_0–168h_ by 25%, without affecting the PK of cetuximab and irinotecan [196]. A previous study of this same triple combination in non-Japanese patients was tolerable, though the PK was unable to be compared to determine if the altered PK is attributable to having Japanese ancestry [200].

**Figure 4:**
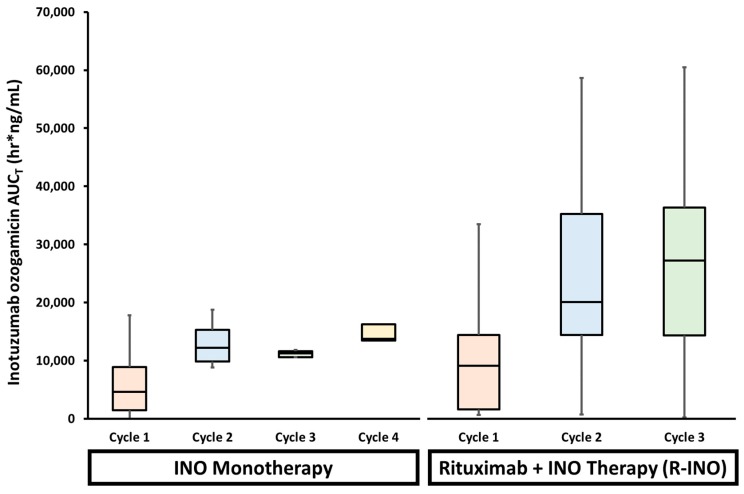
Serum inotuzumab ozogamicin (INO) exposures (AUC_T_; cycle one in orange, cycle two in blue, cycle three in green, and cycle four in yellow) in patients receiving INO monotherapy or in combination with rituximab (R-INO). R-INO combination therapy demonstrated a higher variability in INO AUC_T_ compared to INO monotherapy. In addition, INO exposure was ~three-fold higher in patients receiving combination therapy compared to monotherapy. These findings suggest that combining two immunotherapeutic antibody agents may saturate shared mechanisms of clearance, thus increasing the likelihood of inter-patient variable exposure.

Inotuzumab ozogamicin (Besponsa^®^; an anti-CD22 ADC; Wyeth Pharmaceuticals LLC, Philadelphia, PA, USA) is used for the treatment of relapsed or refractory B-cell precursor acute lymphoblastic leukemia (ALL). To evaluate potential changes in the PK and efficacy of inotuzumab ozogamicin used in combination with rituximab (R-INO), a phase I/II study in relapsed/refractory B cell lymphoma patients was performed [201]. The MTD of inotuzumab ozogamicin in combination with rituximab (administered on day 1: 375 mg/m^2^) was confirmed to be the same as that for single-agent inotuzumab ozogamicin (administered on day 2: 1.8 mg/m^2^ every four weeks). The mean serum ADC values after the administration of inotuzumab ozogamicin alone were similar to R-INO therapy on cycle 1 (~4,500 vs. 9,000 h.ng/mL, respectively), though both displayed significant variability (Figure 4). However, by the third cycle, the mean serum AUC exposure of ADC was three times greater in patients administered R-INO compared to inotuzumab ozogamicin alone (~10,000 vs. 28,000 h.ng/mL, respectively) and R-INO displayed higher inter-patient variability (Figure 4). These data suggest that combining additional immunotherapy agents with inotuzumab ozogamicin reduces the clearance and increases the exposure of inotuzumab ozogamicin by ~three-fold. The altered PK of inotuzumab ozogamicin is consistent with the theory that combining mAbs saturates the uptake of these agents by immune cells, which then decreases the overall clearance and increases the toxicity of the agents.

Compared to numerous studies of small molecule drug interactions, there are scarce evaluations of how biologics (including therapeutic antibodies) can alter the PK and PD of other ADCs. Reports detailing the combined use ADCs with other antibodies only recently begun to be published, and mainly focus on the discovery of synergistic effects within preclinical models. Thus, while data specific to ADCs is lacking, the field has in general observed an increase in knowledge about DDIs after the administration of multiple therapeutic proteins or with traditional cytotoxic agents which would imply that this is an important interaction to evaluate during agent screening and selection, preclinical studies, and clinical trials.

### 5.2. Ocular Toxicity

Ocular toxicity events have been described in human clinical trials in conjunction with the administration of ADCs. This is due to the eye being uniquely susceptible to potential toxicities, including having significant vascular access (delivering a robust blood supply), the presence of numerous and rapidly dividing cell types, and a variety of surface receptors on these cells [202,203]. Due to the variety in potential delivery and interaction with the eye, the occurrence and severity have been variable, with reactions varying from minor irritation to vision-threatening events [202,203]. However, toxicities have also been observed for ADCs against different targets not expressed in the eye, such as CD19, folate receptors, CD70, and numerous others-complicating the prediction of such toxicity events [204,205,206,207,208,209,210]. Ocular events typically improved or resolved after ADC administration ceased [211].

A recent review of the literature sought to determine common factors among the reports of adverse ocular events of ADCs within human trials [211]. After a systematic review, a total of 22 studies were found citing ocular toxicities suspected to be due to ADC administration. Ocular events were associated with a variety of tissues in the eye and most events were not severe (i.e., ≤grade 2 based on CTCAE) or resultant in a dose-limiting toxicity. This analysis showed that ADCs containing either MMAF or DM4 (and their respective linkers) were potentially more prone to ocular adverse events and a lesser percentage with vcMMAE drug linkers. However, as the reporting of ocular events was inconsistent among trials and studies, the underlying toxicological mechanism(s) has/have still not been identified and additional studies are necessary in order to design future ADCs to avoid this toxicity.

## 6. Preclinical Model Considerations

The advent of immunotherapy and ADCs has changed the landscape of treating patients with cancer, but the fact remains that a subset of patients either do not respond or experience severe toxicities. Experts in the field agree that the next goal is to expand the number of patients who can benefit from ADCs and other immunotherapies, raising questions as to which host factors may dictate the divergent outcomes observed. However, one of the primary obstacles in the development and validation of biomarkers or predictive studies to overcome this issue is the use of animal models that fail to mimic (or fail to fully mimic) the human condition. As practical and ethical concerns surround human subject use in cancer, preclinical animal models will remain a cornerstone in the safety and efficacy studies of translational cancer research. Despite this, there is a clear knowledge gap in understanding the variation in the numerous models available as the successful translation from animal models into human trials has been reported to be, on average, ~8% [212,213,214,215]. While several of these human trial failures can be linked to study design issues, a significant portion can be traced back to mechanisms of the drugs being tested in preclinical studies [216]. 

It is also important to understand that ADC development is a complex process. While many of the limitations and considerations for ADCs are the same for “naked” antibodies, special consideration needs to be taken for ADCs as they must preferentially bind to tumors in order to be internalized to release their cytotoxic drug. Despite the large arsenal of in vitro and in vivo assays, there is a constant demand for new tools that are more predictive of clinical outcomes in patients. 

### 6.1. General Limitations in Preclinical Tumor Implantation

It is important to note that xenograft models have been utilized extensively in the selection and development of numerous anti-cancer agents, including ADCs [217,218]. In addition, these xenograft models have demonstrated a correlation between animal model activity and clinical trials, and thus have been considered clinically-relevant tools [217]. However, the largest limitation of these models is that they do not emulate all stages of cancer progression [219,220,221,222]. This is because implanted xenografts tend to grow at a higher proliferative capacity in animals compared to what is observed in human patients [221,222,223,224,225]. Furthermore, the method of implantation (flank versus orthotopic) and the selection of tumor cell line utilized are important factors, with a significant impact on tumor microenvironment changes [226]. Ultimately, orthotopic models should be utilized where possible because they are more clinically relevant due to the natural anatomic location, mimicking more realistic tumor growth, and thus may be more predictive [226]. Orthotopic implantation can be made further relevant by using patient-derived xenograft (PDX) models, maintaining unique genetic and tissue architectures of the malignancy. While orthotopic implants of PDX models are not commonly employed, these models could establish more predictive outcomes or provide additional insights into small subtypes due to retained unique cellular characteristics. 

### 6.2. Differences in Clinically-Relevant Covariates

**Antigen presentation:** ADCs are targeted agents, thus the chosen preclinical model should express the same target antigen that binds with a similar affinity as within patients. To complicate this further, binding affinities should also be evaluated across multiple tissues (such as by IHC) expressing the target to evaluate a model’s ability to fully emulate human disposition. Finally, ADCs can cause both on-target and off-target toxicities, depending on antigen binding [179,189]. While on-target cytotoxicity may present from internalization by antigen-expressing normal cells (i.e., non-tumor cells), off-target toxicities primarily result from the premature release of the ADC’s cytotoxic drug (via instability, degradation, or internalization by normal cells) [179,189,227]. The more similar the antigen presentation within the model, the better the prediction of the rates and incidence of both on- and off-target toxicities. 

**Age:** A benefit and common use of immunotherapies is in an older population (>60 years old), where traditional small molecule chemotherapy may be too toxic or not as well tolerated [59,228,229]. However, numerous clinical trials of ipilimumab and nivolumab have demonstrated that older patients were at a higher risk of treatment-related toxicities [230]. The immune system undergoes numerous changes as a human ages, so it is argued that age should also be evaluated in preclinical studies by using older animals. As TNF and IL-6 production by macrophages appears to increase with age, and TNF has been linked as a dominant cytokine in regulating immunotherapy toxicities, older patients and models may demonstrate an increased susceptibility to immune-related reactions or annul anti-tumor effects due to deleterious inflammation [231,232,233]. However, most preclinical species utilized in drug studies tend to be younger. For instance, as mice age, they experience chronic inflammation and increased adiposity similar to humans [231,234]. These age effects were also shown to be responsible for immunotherapy-related toxicities in mice older than nine months, but these toxicities were absent in mice less than six months old [231]. While studies continue to be performed in a young preclinical population, their continued use for predicting efficacy and toxicities in a majority human population is important, though an additional older animal cohort can be justified. 

**Body habitus:** It has long been reported that obese individuals demonstrate an altered immune status compared to non-overweight individuals, primarily believed to be driven by chronic inflammation and increased adiposity (in both humans and mouse models) [235,236,237,238,239]. While the precise mechanism(s) connecting obesity-induced inflammation and immune status changes has/have not fully been determined, the effect on the immune system is evident. Studies have demonstrated the decreased proliferation and effector functions of T cells in obese mice that are not caused by intrinsic T cell defects [237,238]. These studies also demonstrated a slightly increased DC frequency in obese mice, though functionally impaired, which could in part contribute to the impaired T cell functionality. The disruption of both T cell and DC function has the capability to impair numerous immune responses in obese individuals and further confound our understanding of tumor response. In addition, the cellular make-up of adipose tissue is predominantly various immune cells (including T cells, B cells, and macrophages) and likely plays a role in the complex inflammation due to obesity [240,241,242]. 

Despite the well-documented incidence of cancer in obese populations, research into the use and safety of immunotherapies in obese patients and models are sparse [243,244]. While clinical trials readily investigate total body weight-based factors in immunotherapy trials without demonstrating a correlation to efficacy or toxicity, more accurate measures of body mass, such as visceral body fat or the waist-to-height ratio, that have not been readily evaluated have been predictive in other non-malignant inflammatory conditions [245]. 

### 6.3. Differences in Immune Cells & Phagocytes In Pre-Clinical Models

While in vitro models provide their own advantages in modeling biological systems, the function of the immune system is complex and requires a network of communication among other immune cells and other cells throughout the body. Thus, it is difficult to recreate these systems in vitro, necessitating the use of model organisms, especially to elucidate immune-mediated reactions. Moreover, unlike traditional chemotherapies, antibody therapeutics can present with unique toxicities due to accumulation within immune cells or organs, which can only be analyzed in vivo. Below are a handful of clinically-relevant variables relating to the immune response to guide the design of preclinical studies of immunotherapies. 

**Immune Reactivity:** To correctly translate results from preclinical animal models to human studies, the diversity of the potential immune reactivity between species, or between inbred strains of the same species, needs to be considered during study design. However, these differences can be wide-ranging. For instance, while numerous agents have been evaluated in safety studies using various in vivo models (e.g., rats, dogs, non-human primates (NHP)), immune-cell directed reactions, such as thrombocytopenia or monocytosis, were more severe in NHP compared to rodent species [54,246]. In addition, it appears that complement-mediated toxicities are also more severe in NHPs [54,246]. Therefore, it is imperative to pick applicable animal species in preclinical analyses and to understand the translation to human patients. 

**Immune Cell Composition & Function:** While many of the main concepts of immunology are retained throughout species, species-related differences of immune cells do exist–primarily in the innate immune response and the ability to maintain oxidative homeostasis. The majority of these analyses have been performed in mice due to their wide-spread use. For instance, the composition of granulocytes (specifically neutrophils) is different between mice and humans, comprising 10–25% of peripheral blood leukocytes in mice, but 50–70% in humans [247]. This is significant not just due to the numerical difference, but because granulocytes are the first cells to reach inflammatory sites and release numerous chemical mediators to incite the host’s defense (including T cells and dendritic cells). The contents of the granules of these cells are also different in mice compared to humans, leading to differences in the overall response to stimuli [248]. Another example is in the polarization of macrophages. The markers that define M1 or M2 macrophages are well-defined by genetic markers and surface receptors, but this identification is less clear in humans due to the altered expression of numerous factors, inconsistent nitrous oxide production, and diminished arginase activity [248,249,250,251,252,253,254]. In addition, studies suggest mediators of macrophage activation, such as IL-4 and IFN-α, are more efficient in inducing macrophage function [254,255]. This may mean that murine models, even humanized models, have limited use in regards to studying the pathogenesis of human inflammatory conditions. However, as most anti-cancer agents focus on a distinct molecular mechanism of action, investigations of these mechanisms in animals are still valuable. 

**Fc-receptor Profiles:** Making comparisons of human disposition of an agent’s disposition using preclinical models is complicated by the fact the FcγR expression profiles vary among preclinical species. For example, mice and humans both express FcγRI on myeloid cells and FcγRIII on NK cells [256,257]. However, an additional receptor type, FcγRIV, an activating Fc-receptor found on neutrophils and MPS cells that binds to IgG2a/b, is found in mice, but is absent within humans [256,257,258]. NHPs also only carry a single FcγRIII gene, which is similar to FcγRIIIa in humans. However, variability in FcγR expression exists even among NHP models. Sooty monkeys express FcγRIII on some lymphocytes, neutrophils, and monocytes; however, macaques and baboons do not express FcγRIII on neutrophils [259]. Additionally, both human and NHP FcγRIII variants interact with IgG1, but human FcγRIII also interacts with IgG3, while NHP variants interact with IgG2 [259]. These data make the selection of particular preclinical models inappropriate to estimate first in human doses and/or treatment-related toxicities.

**FcRn:** Some of the first antibody therapeutics were fully murine antibodies and demonstrated short half-lives (ranging only one to two days) when administered to humans [260]. Several prior reviews have examined how Fc-region mutations affect the IgG-FcRn interaction and it direct relation to differences in the observed half-life of antibody agents [172,261,262,263]. Specifically, five notable mutations have been extensively reviewed for their ability to extend antibody serum half-life [264,265]. However, studies also exist contradicting these results, raising questions on the interaction between FcRn and in vivo clearance [176,266,267]. Differences in FcRn-IgG interactions among species have been assessed to determine their relevance in particular preclinical models, particularly mice. Human IgG antibodies bind with a stronger affinity (~15-fold greater) to murine FcRn, resulting in a slower clearance/increased half-life in mice, which makes mice poor predictors of human clearance in allometric scaling equations [266,268,269,270]. While human FcRn has been demonstrated to bind to human, rabbit, and guinea pig IgGs, it lacks the ability to efficiently bind mouse, rat, sheep, and cow IgG [271]. In contrast, murine FcRn can bind to IgGs from all of these species [271]. In addition, the affinity of murine FcRn for human IgG is greater than that of the murine analog, leading to limitations in evaluating the PK and efficacy of human mAbs in preclinical mouse models [272]. This indicates that preclinical PK studies should rely on genetically engineered mouse models (GEMMs; e.g., transgenic FcRn models), though more efficient high-throughput comparisons using in vitro or ex vivo samples would be valuable [272,273]. High-throughput methods to increase PK/PD analyses would be advantageous, especially as it has been implied that roughly 15% of phase I studies fail due to an unfavorable pharmacologic disposition [274].

**Chemical Mediators of Immunity:** As chemokine effects have been observed to correlate with altered PK disposition in animals, it is valuable to use both animal species and tumor models that emulate a similar cytokine/chemokine response to stimulation to what would occur in patients [128]. Such studies emphasize the role and bi-directional relationship between immune response and alteration of the tumor microenvironment that can alter PK/PD disposition due to immune cell driven activity. These data also highlight the need to consider chemokine effects during the development and validation of animal models. Finally, studies have also shown how chemokine expression may be different between species, such as the case of CCL2 and CCL5 between mice and humans: CCL2 levels are higher than CCL5 in mice, whereas CCL5 levels are higher in humans [128]. It is important to match immune-mediating signaling molecules and activity when utilizing models to account for possible variability observed in immunotherapy PK and PD.

### 6.4. Prediction of Human ADC PK Using Allometry

During drug development, allometric scaling provides a practical approach to predict the PK profile of drugs in a preclinical model species, particularly in the absence of prior drug experience in that species. The assumption for using allometric scaling among species is that there are similarities (including biochemical, physiological, and anatomical) among animals that simple mathematical models can evaluate [275]. Because of this, allometry is also most commonly used in determining the first human dose when an agent enters phase I clinical trials [276]. In addition, several important PK parameters, including CL, volume of distribution (Vd), and half-life (t_1/2, elim_), are regularly predicted among preclinical species and from preclinical models to humans. Traditionally, body weight has been used as a criterion for the extrapolation of drug dose from animals to humans in cancer chemotherapy. On the other hand, the FDA currently recommends the use of body surface area (BSA) with an exponent of 0.67 to scale doses across species for small molecule anti-cancer agents [277]. However, since BSA is not directly measured with allometric equations, and due to the existence of multiple different equations to calculate BSA (e.g., DuBois, Haycock, Gehan), it has been argued that a simple mathematical function of body weight provides similar advantages to using BSA [278,279].

Studies reporting on the prediction of human ADC PK have not been widely published, but this is likely due to the limited clinical experience of the later-stage ADCs that have entered the clinic. Compared to traditional small molecule drugs, toxicology studies of mAb/ADCs need to occur within models that ideally express the target antigen at the same/similar levels and invoke a similar response as would occur in a human [280]. This can limit the number and vary the models available for use, depending on the agent under investigation. Rats and dogs are the most widely utilized preclinical models for toxicokinetic studies [281], though several mAbs have only been evaluated in non-human primates (NHP) because it is the only species that meets the agent’s pharmacologic requirements [280]. Typically, this results in toxicology studies being performed in cynomolgus monkeys as they require less agent for dosing (due to their smaller size in comparison to a rhesus monkey or baboon), and have the most history of use in immunotoxicology and reproductive testing of human mAbs [280]. Alternatively, human antigen transgenic mice may also be used, allowing for a concurrent assessment of agent-related toxicities and local tolerance effects. Such models should be validated before use to ensure that the antigen transgene is expressed on the same cells/tissues and at similar levels in the mouse-human model. In addition, problems in the translation of results from preclinical models to humans have been an issue for mAb agents. Two such examples of species-specific increased CL rates have been described previously by Vugmeyster et al. in monkeys and by Bumbaca et al. in mice [282,283]. 

As a conjugate, the overall ADC PK (as measured by total antibody or conjugated antibody) is primarily going to be driven by the antibody moiety, not the cytotoxic drug. The mechanisms impacting mAb/ADC clearance are comparable between humans and NHP, making species such as cynomolgus monkeys a common preclinical model for testing. Compared to small molecule agents, the estimation of clearance using non-human primate data employing an allometric scaling exponent of 0.85 is suggested [284,285]. Of note, these recommendations are made based on the antibody component guiding the scaling and may not taking into account changes in ADC disposition due to a cytotoxic drug’s characteristics (e.g., increased hydrophobicity, tertiary structure alterations) [53]. Furthermore, this method aids in predicting human antibody PK parameters, but is often difficult in predicting the cytotoxic drug’s (i.e., released/free drug) parameters or disposition. As mentioned earlier, brentuximab vedotin experienced higher released MMAE plasma levels in patients that were not predicted in cynomolgus monkeys, resulting in serious side effects and its removal from the market. Ultimately, additional studies on allometry of ADCs are needed, with a focus on the mechanistic/catabolic differences among species. 

### 6.5. Alternative Strategies: Humans as a Model?

While the issues with preclinical models continue to be deliberated, others have argued that we should bypass the expenditure of resources towards animal models and evaluate therapies directly in humans. In 2007, both the FDA and EMA published guidelines introducing this idea of a “Phase 0” trial, where ‘micro-doses’ of agent are administered to patients [286]. While these trials utilize agents at doses roughly 1/100th of the therapeutic dose, safety is not likely to be compromised. However, valuable data can be collected, including drug distribution and metabolism, and confirm the targeted agent mechanism of action (i.e., level of both targeted and non-specific exposure). Within a small population of patients where agents can be directly tested, key information can be gathered to determine if the drug behaves as expected and is worth additional investment/resources to continue into official human trials. However, as very low doses are utilized, a very sensitive analytical method may need to be developed to detect low drug levels in the body. 

## 7. Conclusions

The field of ADC research and choice of ADCs available have continued to grow rapidly within clinical trials and continue to prove their therapeutic value. However, considerable challenges persist. While the development of ADCs is an evolving field, the rapidly accumulating experience and application of ADCs will become increasingly important for informing rational development and design. In addition, the use of new high-throughput screening platforms with predictive biomarkers that are less time-intensive (such as optical imaging or IHC methods) will be a marked improvement to ensure that patients receive therapies targeted towards their own malignancy and thus receive the most benefit from targeted therapies.

Despite the significant advancements in our understanding of the PK and PD of antibody compounds, an improved understanding of how the safety and efficacy of these agents are affected by individual mechanistic aspects is needed, especially the bi-directional interaction with the innate immune system. Both the efficacy and toxicity associated with ADCs rely on numerous factors, including those involved in the construction of the ADC itself along with patient-specific features. However, mAb and ADC therapies appear to present new toxicities that are related with either an increased distribution to specific organs, like the liver and eyes, or toxicities linked to components of the carrier. Preclinical studies of ADCs in preclinical models can further be complicated by the complex interactions and differences between host immune systems and mechanisms, altering the distribution of proteins. A culmination of these animal studies highlights the difficulty in circumventing many of the drawbacks of their use, but without viable synthetic or cost-effective strategies, these models need to be understood and characterized to highlight appropriate translation of results.

Overall, the pharmacology of mAbs and ADCs is multifaceted. A number of challenges remain to optimize ADC therapies before their use can elevate the field, such as the homogenization of DAR populations, developing methods to improve limited and variable tumor penetration, and the development of resistance. In addition, while early success of the first FDA-approved ADCs (Kadcyla and Adcetris) has emboldened the approval of three additional ADC agents, there is still much to learn about the clinical applications of antibody-based therapy. Furthermore, additional research into value-added treatments and enhanced outcomes based on personal phenotypes using immunotherapies is warranted. Further areas of investigation that can aid in our understanding of these agents include: validation and characterization of commonly utilized preclinical models, cellular function, analysis of patient covariates affecting the immune system (age, body habitus), more robust and sensitive PK methods for evaluating both cytotoxic drugs and antibody-carriers, and combining PK and PD data with phenotypic biomarkers of innate immunity and other systems with appropriate preclinical models. 

## Figures and Tables

**Table 1:** Antibody–drug conjugates approved and under investigation (Phase II or higher).

**Generic Name**	**Brand Name**	**Manufacturer**	**Highest Phase of Studies Open**	**Number of Open Studies**	**Target Antigen**	**Linker**	**Payload**	**Indications**
Moxetumomab pasudotox-tdfk	Lumoxiti	AstraZeneca	Approved–2018	15	CD22	Cleavable (disulfide)	*Pseudomonas* exotoxin A	Hematological
Gemtuzumab ozogamicin	Mylotarg	Pfizer	Approved–2017	83	CD33	Cleavable (acid labile)	Calicheamicin	Hematological
Inotuzumab ozogamicin	Besponsa	Pfizer	Approved–2017	32	CD22	Cleavable (acid labile)	Calicheamicin	Hematological
Trastuzumab emtansine	Kadcyla	Genentech	Approved–2013	85	HER2	Non-cleavable	DM1	Solid
Brentuximab vedotin	Adcetris	Seattle Genetics	Approved–2011	143	CD30	Cleavable (protease)	MMAE	Hematological
**Generic Name**	**Investigational Name**	**Manufacturer**	**Highest Phase of Studies Open**	**Number of Open Studies**	**Target Antigen**	**Linker**	**Payload**	**Indications**
Depatuxizumab mafodotin	ABT-414	Abbvie	III	4	EGFR	Non-cleavable	MMAF	Solid
Enfortumab vedotin	ASG-22CE	Astellas Pharma	III	5	Nectin 4	Cleavable (protease)	MMAE	Solid
Margetuximab	MGAH22	MacroGenics	III	3	HER2	Cleavable (thioether)	DM1	Solid
Mirvetuximab soravtansine	IMGN-853	ImmunoGen	III	6	FOLRI 1	Cleavable (disulfide)	DM4	Solid
Polatuzumab vedotin	DCDS-4501A	Genentech	III	9	CD79b	Cleavable (protease)	MMAE	Hematological
Rovalpituzumab tesirine	SC0001-SCX	Stemcentrx	III	9	DLL3	Cleavable (protease)	SCX	Solid
Sacituzumab govitecan	IMMU-132	Immunomedics	III	4	TROP2 EGP1	Cleavable (acid labile)	SN-38	Solid
Trastuzumab deruxtecan	DS-8201	Daiichi Sankyo Inc.	III	11	ERBB2	Cleavable (Protease)	Topoisomerase I inhibitor	Solid
Trastuzumab duocarmazine	SYD985	Synthon Biopharmaceuticals	III	2	ERBB2	Cleavable (Protease)	Duocarmycin	Solid
PSMA-PyL	18F-DCFPyL	Progenics	II/III	27	Fluorinated PSMA	Cleavable (Protease)	MMAE	Solid
-	AGS-16C3F	Agensys	II	1	AGS-16/ENPP3	Non-cleavable	MMAF	Solid
Anetumab Ravtansine	BAY 94-9343	Bayer Healthcare	II	11	Mesothelin	Cleavable (disulfide)	DM4	Solid
Labetuzumab govitecan	IMMU-130	Immunomedics	II	2	CEACAM5	Cleavable (acid labile)	SN-38	Solid
Tisotumab Vedotin	HuMax-TF	Genmab Seattle Genetics	II	3	Tissue Factor	Cleavable (disulfide)	MMAE	Solid
-	CX-2009	Cytomx	I/II	1	CD166	Cleavable (protease)	DM4	Solid
Enapotamab vedotin	HuMax-AXL	Genmab	I/II	1	AXL	Cleavable (protease)	MMAE	Solid
Indatuximab ravtansine	BT-062	Biotest	I/II	1	CD138	Cleavable (disulfide)	DM4	Hematological
Pinatuzumab vedotin	DCDT-2980S	Genentech	I/II	1	CD22	Cleavable (protease)	MMAE	Hematological

Abbreviations: MMAE, monomethyl auristatin E; DM1, mertansine; DM4, ravtansine; MMAF, monomethyl auristatin F. Studies open (i.e., active) as of October 2018 on ClinicalTrials.gov.

**Table 2:** FDA-approved, non-conjugated monoclonal antibodies for therapeutic use.

Generic Name	Brand Name	Type of Antibody	Antibody Isotype	Target Antigen
**Oncology**				
Nivolumab	Opdivo	Human	IgG4	PD-1R
Daratumumab	Darzalex	Human	IgG1	CD38
Ofatumumab	Arzerra	Human	IgG1	CD20
Durvalumab	Imfinzi	Human	IgG1	PD-L1
Ipilimumab	Yervoy	Human	IgG1	CTLA-4
Necitumumab	Portrazza	Human	IgG1	EGFR
Ramucirumab	Cyramza	Human	IgG1	VEGFR2
Olaratumab	Lartruvo	Human	IgG1	PDGFRa
Panitumumab	Vectibix	Human	IgG2	EGFR
Avelumab	Bavencio	Human	IgG1	PD-L1
Cemiplimab-rwlc	Libtayo	Human	IgG4	PD-1
Atezolizumab	Tecentriq	Humanized	IgG1	PD-L1
Elotuzumab	Empliciti	Humanized	IgG1	SLAMF7
Obinutuzumab	Gazyva	Humanized	IgG1	CD20
Pembrolizumab	Keytruda	Humanized	IgG4	PD-1R
Bevacizumab	Avastin	Humanized	IgG1	VEGF
Bevacizumab-awwb	Mvasi	Humanized	IgG1	VEGF
Pertuzumab	Perjeta	Humanized	IgG1	HER2
Alemtuzumab	Campath Lemtrada	Humanized	IgG1	CD52
Trastuzumab	Herceptin	Humanized	IgG1	HER2
Trastuzumab-dkst	Ogivri	Humanized	IgG1	HER2
Trastuzumab-pkrb	Herzuma	Humanized	IgG1	HER2
Blinatumomab	Blincyto	Humanized	IgG1	CD19
Rituximab/Hyaluronidase	Rituxan Hycela	Chimeric	IgG1	CD20
Rituximab	Rituxan	Chimeric	IgG1	CD20
Rituximab-abbs	Truxima	Chimeric	IgG1	CD20
Dinutuximab	Unituxin	Chimeric	IgG1	disialoganglioside GD2
Cetuximab	Erbitux	Chimeric	IgG1	EGFR
**Inflammatory Diseases**				
Ustekinumab	Stelara	Human	IgG1	IL-12/IL-23
Secukinumab	Cosentyx	Human	IgG1	IL6
Belimumab	Benlysta	Human	IgG1	BLyS
Guselkumab	Tremfya	Human	IgG1	IL23
Adalimumab	Humira	Human	IgG1	TNFa
Adalimumab-atto	Amjevita	Human	IgG1	TNFa
Adalimumab-adbm	Cyltezo	Human	IgG1	TNFa
Adalimumab-adaz	Hyrimoz	Human	IgG1	TNFa
Golimumab	Simponi	Human	IgG1	TNFa
Sarilumab	Kevzara	Human	IgG1	IL6R
Dupilumab	Dupixent	Human	IgG4	IL4Ra
Brodalumab	Siliq	Human	IgG2	IL-17a
Vedolizumab	Entyvio	Humanized	IgG1	a4b7 integrin
Certolizumab pegol	Cimzia	Humanized	Fab	TNFa
Ixekizumab	Taltz	Humanized	IgG4	IL-17a
Tocilizumab	Actemra	Humanized	IgG1	IL-6 receptor
Natalizumab	Tysarbi	Humanized	IgG4	a4-integrin
Efalizumab	Raptiva	Humanized	IgG1	CD11a
Tildrakizumab-asmn	Ilumya	Humanized	IgG1	IL-23
Infliximab	Remicade	Chimeric	IgG1	TNFa
Infliximab-abda	Renflexis	Chimeric	IgG1	TNFa
Infliximab-dyyb	Inflectra	Chimeric	IgG1	TNFa
Infliximab-qbtx	Ixifi	Chimeric	IgG1	TNFa
**Organ Transplant**				
Daclizumab	Zinbryta	Humanized	IgG1	CD25
Basiliximab	Simulect	Chimeric	IgG1	CD25
Muromonab-CD3	Orthoclone-OKT3	Murine	IgG2a	CD3
**Miscellaneous Conditions**				
Canakinumab	Ilaris	Human	IgG1	IL1B
Denosumab	Prolia Xgeva	Human	IgG2	RANKL
Bezlotoxumab	Zinplava	Human	IgG1	C. difficile toxin B
Alirocumab	Praluent	Human	IgG1	PCSK9
Evolocumab	Repatha	Human	IgG2	PCSK9
Erenumab-aooe	Aimovig	Human	IgG2	CGRP
Burosumab-twza	Crysvita	Human	IgG1	FGF23
Emapalumab-lzsg	Gamifant	Human	IgG1	IFNg
Raxibacumab	Raxibacumab	Human	IgG1	B. anthracis toxin
Lanadelumab-flyo	Takhzyro	Human	IgG1	Kallikrein
Ocrelizumab	Ocrevus	Humanized	gG1	CD20
Omalizumab	Xolair	Humanized	IgG1	IgE
Reslizumab	Cinqair	Humanized	IgG4	IL5
Daclizumab	Zinbryta	Humanized	IgG1	IL2R
Mepolizumab	Nucala	Humanized	IgG1	IL5
Ranibizumab	Lucentis	Humanized	IgG1	VEGFR1, VEGFR2
Idarucizumab	Praxabind	Humanized	IgG1	Dabigatran
Fremanezumab-vfrm	Ajovy	Humanized	IgG2	CGRP
Galcanezumab-gnim	Emgality	Humanized	IgG4	CGRP
Benralizumab	Fasenra	Humanized	IgG1	IL-5Ra
Emicizumab-kxwh	Hemlibra	Humanized	IgG4	Factor IXa & Factor X
Mogamulizumab-kpkc	Poteligeo	Humanized	IgG1	CCR4
Mepolizumab	Nucala	Humanized	IgG1	IL-5
Ibalizumab-uiyk	Trogarzo	Humanized	IgG4	HIV-1
Obiltoxaximab	Anthem	Chimeric	IgG1	Anthrax toxin
Siltuximab	Sylvant	Chimeric	IgG1	IL-6

## Abbreviations

ADA	anti-drug antibodies
ADC	antibody-drug conjugate
ADCC	antibody-dependent cell-mediated cytotoxicity
ALL	acute lymphoblastic leukemia
AML	acute myeloid leukemia
AUC	area under the curve
BBB	blood-brain barrier
BSA	body surface area
CDC	complement-dependent cytotoxicity
CMA	carrier-mediated agent
CL	clearance
DAR	drug–antibody ratio
DDI	drug–drug interaction
DM1	mertansine
DM4	ravtansine
ESRD	end stage renal disease
Fabs	Fab fragments
FcγR	Fc-gamma receptors
FcRn	neonatal Fc receptor
HGF	hepatocyte growth factor
iv	intravenously
KO	knockout
mAb	monoclonal antibody
MMAE	monomethyl auristatin E
MMAF	monomethyl auristatin F
MPS	mononuclear phagocyte system
NHP	non-human primate
NSCLC	non-small cell lung cancer
NP	nanoparticle
PD	pharmacodynamic
PDX	patient-derived xenografts
PEG	polyethylene glycol
PFS	progression-free survival
pI	isoelectric point
PK	pharmacokinetic
SABV	“sex as a biologic variable”
sc	subcutaneously
scFv	single chain variable fragment
TAM	tumor-associated macrophage
WT	wild type
